# Remarks upon Menstruation—Physiological and Pathological

**Published:** 1859-04

**Authors:** Hayden Coe

**Affiliations:** Atlanta, Ga.


### ARTICLE II.

*Remarks upon Menstruation—Physiological and Pathological.*

By Hayden Coe, M. D., Atlanta, Ga.

Much discussion has taken place respecting the causes, nature and purposes of the menstrual flow ; and it has been discussed with so much ability, that it may appear presumption in me to add anything more on this subject. But in a pathological or practical point of view, it is of vast importance that the nature and purposes of the menstrual flow be well understood. Formally, the profession unanimously supposed, that the menstrul flow was necessary to rid the system of a fluid, which, if retained, would act deliterious to the system—that is, act as a poison—and in all cases when it was arrested, it was essential that it be restored, in order to relieve the system of a morbific agent, or poison ; nor is it without its advocates at the present time ; and we constantly hear men speak of disease arising from the arrest of the menstrual flow, and it must be restored, in order to relieve the patient.

This is the prevailing notion of the present time, particularly with the people, than which, nothing can be more erroneous. They mistake the effect for the cause. It is true, if we were to view the menstrual flow as a secretion, and necessary to remove a fluid which would act as a poison, if retained, as if the case with a retention of urine or bile in the blood, then the above theory of the disease would be correct. That it is a secretion, we think it cannot be sustained, either by the anatomy or physiological action of the organs concerned, or the nature of the fluid discharged.

The uterus from which the menstrual fluid flows, is composed of three coats, the serous, the muscular and mucous. The serous coat is a portion of the peritoneum, reflected over the uterus ; the muscular coat is composed of three layers, the middle is much the thickest, and is the larger of the muscular coat, in which the numerous blood vessels ramify, which become so much enlarged during pregnancy. The mucous coat, like all other mucous membranes, is lined by an epithelium, composed of epithelial cells, for the secretion of a fluid for the protection of the surface of these delicate membranes. It is true, that Todd and Boman speak of a few mucous folicles found in the mucous membrane of the uterus, but they say that they only contain a little whitish mucous. So we find no glandular apparatus in the anatomy of the uterus, for secreting the menstrual fluid.—In all instances where we have a fluid secreted for any special purpose, we have glands composed of folicles, or cul-de-sac, with ducts of greater or less length, which folicles are situated on a basement membrane; each folicle has the power of generating cells, which cells have the power of selecting from the blood the constituents of the fluid to be secreted; and of depositing it in the folicles to be discharged through their ducts. The fluid thus secreted cannot be obtained from the blood by any other means. The fluid secreted, in all cases, is essentially different from the constituents of the blood; as the bile, the urine, gastric juice, the sebaceous secretion of the folicles of the skin, the secretion of the wax of the ear, &c., none of which can be detected in the blood in its normal state.

We deem it unnecessary to say anything in reference to the anatomy of the ovaria, although the flow of the menstrual fluid is indirectly dependent on the presence of these organs, in a healthy condition; yet it is very evident, that the fluid does not flow from the ovaria but from the uterus. What are the functions that the ovaria and uterus are required or expected to perform? Is it to prepare and mature an ovum for fecundation, and bring about a state or condition of the system, by which fecundation would be most likely to be accomplished? For this flow is not confined to the human female, but something similar occurs in a large portion of the animal kingdom; as we see its effects in many animals during the period termed heat. It controls more or less all the actions in animals, during that period, and its effect is also manifest in the human female.

How is this condition, of not only the organs of generation, but the whole system, brought about? It is in this way, every organ, or tissue, is endowed with a principle which we call excitability, peculiar to itself. The organs of generationas, well as other organs, have their own peculiar excitability. This excitability is maintained, when furnished with nutritious fluid, or histogenetic and calorific material; but if deprived of this material, this excitability is diminished. If this excitability is increased, it must be by furnishing it with an extra amount of this fluid, blood, or histogenetic material. During the menstrual flow in the female, or heat in the animal, this is just what occurs; the principal organs of generation are furnished with an extra amount of blood, consequently an extra amount of histogenetic and calorific material, which is consumed, increasing the peculiar excitability of the organs of generation; they are actually in an exalted state; their normal functions, so to speak, are all increased, so at this period you not only have the ovum matured and prepared for fecundation as a consequence, but have the system placid—in a condition in which it is much more likely to take place than at any other period.

But in the event that fecundation does not take place, it is essential that this exalted condition be removed. How can this be done? Only by removing the cause. Therefore we must remove the extra amount of blood, or histogenetic and calorific material; and as the caliber of the veins are not increased, it cannot be removed in that way, consequently it must be removed by escaping from the mucous membrane of the uterus, as we see it does. It is evident, that unless these organs were brought into this high state of excitement, the ovum would never be prepared for fecundation, nor would the system be in a condition by which it would be accomplished. And this excitement could not be produced by any other means than furnishing these organs with an excess of histogenetic and calorific material. But it may be said, that if conception takes place, then you would have an excitement, or hemorrhage, for nine months. Not so, for then we have a use for our histogenetic and calorific material; it is converted into tissue. The unimpregnated uterus, which is not more than two and a half inches long, one and a half or two broad, is at the full term of gestation, twelve or thirteen inches long, and eight or nine broad ; and this excess of growth has taken place in the short space of nine months.

It will not be necessary for me to give the constituents of the menstrual fluid—they are too well known—neither will it be necessary to compare the constituents of this fluid with the constituents of the blood, to prove that it is blood. It is well known, that they only differ from the blood in a want of fibrine, which is destroyed by the mucous, and even that is present to some extent, when the flow is free, or more than the usual amount. If the constituents of the menstrual fluid are the same as the blood, this being retained in the blood could not produce disease; they could not act as a poison, for it is actually blood itself. But disease set up in these organs arrests its flow, and a little exposure to cold, or anything else that makes a decided impression on the system when these organs are in this high state of excitement, will produce disease. But in our treatment, we only have to subdue the disease, and the flow will return at the proper time. Let us banish the idea from our minds, of its being a secretion, and when ever we are called on, as we frequently are, to restore the secretion, or as it is more commonly called, menstrual flow, tell our patients, that we will endeavor to remove the disease and then the menstrul flow will return without any help from us.

